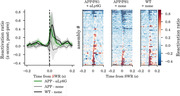# Rescue of impaired hippocampal consolidation in the APP/PS1 mouse model of Alzheimer’s disease after increasing cerebral blood flow

**DOI:** 10.1002/alz.088977

**Published:** 2025-01-03

**Authors:** Laura E Berkowitz, Ralitsa Todorova, Ryan E Harvey, Jean‐Luc Shimizu, Xuelai Dong, Azahara Oliva, Antonio Fernandez‐Ruiz, Nozomi Nishimura, Chris B Schaffer

**Affiliations:** ^1^ Cornell University, Ithaca, NY USA

## Abstract

**Background:**

Alzheimer’s disease (AD) manifests with early spatial memory impairment and is linked to the degeneration of hippocampal circuits. Hippocampal sharp wave ripples (SWRs) are high‐frequency population‐burst events that coordinate the reactivation of neural assemblies (groups of neurons that become correlated in their firing patterns during learning) in post‐learning sleep, which is the neural basis of memory consolidation. SWRs are reduced in the APP/PS1 mouse model of AD‐like pathology. Previously, we showed that cerebral blood flow (CBF) decreases and memory deficits were rescued following treatment with anti‐Ly6G antibodies. Here, we examine the potential normalization of hippocampal circuit activity with CBF increase.

**Method:**

Male, 7‐14‐month‐old APP/PS1 mice and wild‐type controls were implanted with 64‐channel silicon probes in hippocampal area CA1. Neural activity was recorded during sleep before and after the exploration of an open field. Putative cell types were identified using feature‐based classification, and neural assemblies were detected using independent component analysis.

**Result:**

APP/PS1 mice had reduced magnitude and duration of assembly reactivation in post‐task sleep SWRs. After treatment with anti‐Ly6G antibodies, which increase CBF and improve memory performance, we found increased reactivation of these assemblies in post‐task sleep SWRs, relative to no‐treatment controls (Figure 1).

**Conclusion:**

We found that increasing CBF normalizes neural mechanisms of memory consolidation that are altered in AD mouse models, supporting the development of treatment approaches to increase CBF in AD.